# The Burden and Psychological Distress of Family Caregivers of Individuals with Autism Spectrum Disorder: A Gender Approach

**DOI:** 10.3390/jcm13102861

**Published:** 2024-05-13

**Authors:** Raquel Herrero, Amelia Díaz, Jesús Zueco

**Affiliations:** 1 Faculty of Psychology, University of Valencia, 46010 Valencia, Spain; rahella@alumni.uv.es (R.H.); amelia.diaz@uv.es (A.D.); 2 Faculty of Pharmacy, University of Valencia, 46100 Burjassot, Spain

**Keywords:** gender, objective burden, subjective burden, psychological distress, caregivers, autism spectrum disorders

## Abstract

**Background/Objectives:** Relatives play the main role as caregivers of autism spectrum disorder (ASD) individuals. Women, specifically mothers, are the majority of caregivers of ASD relatives. In addition, the literature on caregivers has shown that women have worse mental health and higher perceived burdens than men. Therefore, the aim of this work was to evaluate the relationships between psychological distress and burden using a gender approach in caregivers of ASD relatives. **Methods**: A cross-sectional design was applied in this study with a convenience sample of 250 caregivers of ASD relatives. Most of them were mothers caring for a child who ranged in age from 1 to 31 years. Sociodemographic variables considered were age, education level, marital status, and relation to the care recipient. Additionally, psychological distress and objective burden, in the form of hours/day caring, and subjective burden, in the form of perceived burden, were analyzed. **Results**: Significant gender differences were found in psychological distress and objective and subjective burden, with women showing higher scores than men. Both types of burden played a serial mediating role between gender and psychological distress. **Conclusions**: The results highlight the important role of gender, with women bearing the high cost of caring for their children with ASD in the form of high objective burden, caring for more hours, and subjective burden, perceiving more burden and showing poorer mental health than men. These results show the need for specific support and intervention programs targeted to women caregivers to reduce burden and improve their mental health.

## 1. Introduction

The American Psychiatric Association’s Diagnostic and Statistical Manual, in its Fifth Edition (DSM-5), provides a clear definition and standardized criteria to diagnose autism spectrum disorder (ASD), defining it as a neurodevelopmental disorder characterized by social-communication deficits, restricted and repetitive interests/behaviors, and unusual sensory responses different from other disorders. People with ASD may have difficulties initiating or responding to social interactions, maintaining eye contact or using gestures, and may also present repetitive motor movements; persistence in the repetition of routines, including verbal or non-verbal behaviors; difficulties with transitioning from one activity to another; or focus on a specific interest with too strong intensity. These characteristics could be detected in the early development periods, but the diagnosis may be confirmed much later [1]. The diagnosis of autism is usually made at 18–24 months, as it is at this age when the symptoms of autism can be distinguished from other developmental disorders, although no conclusive behavioral or diagnostic markers for ASD have been identified in children aged less than 12 months [2]

Different reviews have been performed on ASD epidemiology in the last two decades, showing increasing prevalence levels. Elsabbagh et al. [3] conducted a worldwide systematic ASD review with data up to 2012 and proposed the median prevalence of pervasive developmental disorder as 6.16/1000 and that of autism disorder as 1/1000, with a higher prevalence in boys than girls. However, studies from other world regions like Eastern Europe and Africa were absent, and most of the studies included only children younger than 18 years of age. Two years later, in 2014, Tsai [4] found a slight increase in the medians, with 6.19/1000 for pervasive development disorder and 1.32/1000 for autism disorder. In 2020, Chiarotti and Venerosi [5] published a review of worldwide prevalence estimates since 2014, not on separate autism and pervasive developmental disorders but on ASD, where the increase and high variability in the rates were highlighted. They found that the lowest rate corresponded to Bangladesh (0.8/1000) and the highest to Japan (93/1000); this high variability depends, among other factors, on methodological aspects, such as whether the data come from administrative records or from interviews with parents or teachers. No estimates were found for the states of Africa or Central or South America. A more recent review, performed in 2022, estimated the worldwide prevalence of ASD at 1/1000, but again, leaving out many low and middle-income countries from the estimation [6,7]. The ratios of boys and girls (male:female) ranged between 3:1 [8] and 5:1 in clinical or school-based population studies [9].

In addition to the core features of autism, some people with autism also present certain comorbidity with other medical conditions. The most prevalent are attention deficit hyperactivity disorder (ADHD), present in one in every three ASD cases, and learning and intellectual disability and anxiety disorder, present in one in every five cases. The prevalence of just a single comorbidity in ASD is at least twice that of individuals without ASD diagnosis, and this has strong repercussions on the quality of life of patients and caregivers [10].

Autistic people show high differences in their abilities/disabilities, varying along a continuum of severity [11]. While some people with ASD have superior levels of intellectual functioning, others present profound impairment. This means that while some can live independently, others have serious difficulties in doing so, needing life-long care and support. Therefore, a significant proportion of people with ASD require family members, especially parents, to provide care for and support them in many essential activities of daily life.

The challenges experienced by caregivers with ASD relatives needing care and support due to autism symptoms and co-occurring conditions are double. First, the difficulties these ASD caregivers face in accessing health care, education, and training for their relatives, and second, the social, financial, and emotional challenges they experience when caregiving for their ASD relative, where mental health and burden have relevant roles [12]. When caregivers caring for a relative with and without ASD were compared, those caring for a relative with ASD presented more mental health problems than those caring for relatives without ASD [13]. In general, they are characterized by high levels of stress and negative state affect, accompanied by depression and anxiety symptoms [14]. Additionally, the challenges and difficulties experienced by these caregivers have a negative impact on the burden shown by these caregivers, presenting usually from mild to severe levels [15].

The burden of caregivers of ASD relatives is an important variable to be assessed in both dimensions—both in terms of the objective burden, which is associated with the observable and tangible impact of caring on the caregiver, such as the time dedicated to caring or the difficulty in the care tasks performed, and in terms of the subjective burden, also referred to as perceived burden, which is related to the emotional consequences of caring, such as the caregiver’s worry, discomfort, or fatigue [16]. According to studies evaluating the burden in ASD caregivers, this burden is higher than that shown by caregivers of children without ASD due to the piling-up demands of the care recipients [17] and higher also than that shown by caregivers of children with schizophrenia [18]. Two recent studies carried out on family caregivers of ASD people, using the Zarit Burden Interview [19] and the levels proposed by Mulud and McCarthy [20], found that 86.6% showed different levels of burden: 49% were in the mild level, 35% were in the moderate level, and 2.5% were in the severe level, with an average score of 41, corresponding to a moderate level [21,22]. Likewise, when the perceived burden was analyzed in two studies with ADS caregiver samples in Turkey, the stronger predictor was depressive symptoms in the caregivers [18,23]. The relation of depressive symptoms with perceived burden seems to show a clear picture with significant and positive relationships [18,21,22,23,24]. Other variables, such as anxiety or low educational level, were significantly associated with the burden of caregiving in Pandey and Shama’s study [21], but education level was not related to perceived burden in the Al-Qahtani one [22].

Several studies have related the severity of autism symptoms with caregivers’ mental health, but whereas some of them found a significant association [25,26], it is not the case in others [27]. Other studies have highlighted the role of co-occurring circumstances in ASD relatives, such as emotional regulation problems and behavior problems, as the most important factor worsening the caregiver’s mental health [27,28]. The Salomone et al. [27] study found that half the caregivers reported mental health difficulties, and the variables associated were the sociodemographic characteristics of higher educational level and lower household income. The most frequent mental health problems found in this type of caregiver were depression symptoms [25,29], anxiety [30,31], stress [25,32], and hassles in daily activities [29]. Variables more strongly associated with a better mental health in ASD caregivers were lower stress [32,33], higher household income [27,34,35], lack of co-occurring difficulties in the relative [27,28] and lower burden, both objective burden [36] in the form of lower hours of caring and subjective burden [18,21,22,23,24] in the form of lower perceived burden. 

In most studies on ASD family caregivers, the sample is made up of the parents of the ASD care recipient; however, in most cases, it is the mother who is the caregiver assessed. In a systematic review and meta-analysis performed by Yorke et al. [28] about the association between emotional and behavioral problems in children with autism spectrum disorder and psychological distress in their parents that included 67 studies, the mother was the only caregiver evaluated in 34 (50.7%). In 2023, Vast et al. [37] analyzed the gender differences in caregiving among families of people with ASD and found significant differences in the type of tasks carried out by men and women, their caregiving roles, their involvement, and their perceived burden. Men undertook tasks and responsibilities outside the house, whilst their role as providers of immediate care was negligible, with low involvement in the daily basic tasks and consequently lower perceived burden. On the other hand, women provided care for their ASD relative in more crucial tasks, including necessary and basic daily activities, most of them inside the house, with a stronger involvement and experiencing higher burden than men. The socialization received by women from an early age determines, in most cases, their gender role, including the care of others and, in many cases, putting the wellbeing of others above their own wellbeing [38]. Therefore, women integrate this role socially assigned to them and take the responsibility of caregiving as a part of their nature [39]. This higher involvement in the caregiving tasks produces higher levels of psychological distress in mothers when compared with fathers [37,40,41], and specifically when depression scales were used, more than one-third of the mothers caring for a child with ASD showed scores higher than the clinical cut-off [42]. Finally, when positive and negative aspects of caregiving were analyzed between men and women, women scored higher in the negative and lower in the positive aspects, whereas men presented the opposite pattern [43]. The studies comparing gender differences when caregiving for a relative with ASD confirmed the gender gap that still exists between men and women when caring for a family member in need of care and support, with women presenting more perceived burden and poorer mental health than men [44,45,46,47,48]. 

In the context of all the above studies on ASD caregivers, there are three aspects that are extremely relevant, and these are gender, burden, and mental health. The present work aims to contribute to the study of the mental health of ASD caregivers, assessing it using the variable psychological distress, broadly defined as a state of emotional suffering characterized by symptoms of depression and anxiety. Additionally, burden is measured in its two dimensions: objective burden is assessed as hours per day caring, and subjective burden is assessed as the perceived burden. The following hypotheses were tested: (1) women caregivers would present a higher burden, both objective and subjective, and psychological distress than men; (2) objective and subjective burden could play a serial mediation role between gender and psychological distress, where gender would be the independent variable, objective burden the first mediator, subjective burden the second mediator, and psychological distress the dependent variable. 

## 2. Materials and Methods

### 2.1. Participants, Design, and Procedure

A convenience sample of 250 caregivers of relatives with the diagnosis of ASD was obtained from ASD centers in Spain. The inclusion criteria were (1) the relative receiving care had been diagnosed with ASD, (2) the relative was living in the community, (3) the caregiver was at least 18 years old, and (4) there was no reading or understanding problem to complete the information requested. Exclusion criteria were (1) being a caregiver of a relative diagnosed with disorders different from ASD, (2) the relative with ASD being institutionalized, and (3) caregivers that did not complete all the information requested or incompletely answered the questionnaires. The ASD diagnostic was performed by the neuropsychologist working in the corresponding ASD center.

The design used is a descriptive cross-sectional design. All participants first signed the informed consent form, and then they were assessed individually, assigning a number to each participant to preserve their anonymity; consequently, no information about the contribution of other members of the family in caregiving was requested. Participation in this study was voluntary, and no reward was given. The sample collection was in person when the participants were residing in proximity to the researchers or by email when they were residing in other cities. In this last case, the information about this study was sent to the directors of the ASD centers with the first author’s email. These directors informed the members of their ASD center, and caregivers who wanted to collaborate in this study were given the questionnaires once the informed consent form was signed. When the questionnaires and information requested were complete, the participants sent them to the first author via email. The collection of data finished after the 12 months given to complete the information requested. The number of ASD centers included was 65, covering all Spanish provinces. Permission to perform this research was obtained from both the Ethical Committee for Scientific Research of the University of Valencia (H1367489852167) and the ASD centers. 

### 2.2. Measures

#### 2.2.1. Sociodemographic Variable and Objective Burden

The sociodemographic variables assessed were care recipient’s age (<3 years, 3–12 years, 13–17 years, and 18–31 years), caregiver´s age, educational level (primary, secondary and university studies), marital status (single/separated/widow and married/with a partner), hours per day caring (<5 h, 5–10 h, 11–15 h and >15 h), and relation with the care recipient (parents and other).

#### 2.2.2. Burden

Zarit’s Caregiver Burden Interview, CBI [19], was used to assess the caregiver’s perceived burden. The 22 items were answered on a five-point scale from zero (never) to four (nearly always). High scores reflected the high perceived burden. When perceived burden was considered as a categorical variable, the percentages of caregivers in each category were obtained using the Mulud and McCarthy [20] different burden levels in the CBI scale: ≤20 no burden; 21–40 mild burden; 41–60 moderate burden; ≥60 severe burden. Cronbach’s *α* was 0.90 in this study. This scale was chosen because it was specifically designed to measure perceived burden in caregivers, and its psychometric properties are adequate for this purpose [12,41].

#### 2.2.3. Psychological Distress

The General Health Questionnaire, GHQ-12 [49], was completed to evaluate psychological distress. The questionnaire is a mental health screening tool used to assess anxiety, depression, and social dysfunction. The 12 items were answered in a four-point response format (0, 1, 2, 3), with higher scores indicating higher psychological distress. To calculate the percentages of caregivers with and without psychological distress, the GHQ Inx ≥ 14 was taken as the threshold [50] with better sensibility (85.5%) and specificity (83.2%). The reliability in this study was adequate (Cronbach’s *α* = 0.87). The psychometric properties of this questionnaire are adequate for the purpose intended [49,50].

### 2.3. Statistical Analysis

The percentages of sociodemographic variables with the addition of hours per day caring as objective burden were compared between men and women using the *χ*^2^ value. Gender differences analyses were performed using the continuous variables of caregiver’s age, perceived burden, and psychological distress through the Student’s *t* and Cohen’s *d*. When perceived burden and psychological distress are used as categorical variables, gender differences were calculated between the corresponding percentages performing the *χ*^2^ value. The relationships between men and women are presented separately for men and for women using Pearson’ *r* for the continuous variables, caregiver’s age, perceived burden, and psychological distress, whereas Spearman’s *rho* was performed for the categorical variable hours per day caring. Finally, based on the previous relations, a mediational analysis was performed to find the mediational role of objective (hours per day caring) and subjective burden (perceived burden) between gender and psychological distress. The mediation performed is based on ordinary least squares regression and the bootstrap method. The statistical significance of the indirect mediating effects of variables upon the bootstrap method is evaluated based on whether the point estimate of the mediating variable is zero within a 95% bias-corrected and accelerated confidence interval (BCaCI). That means that a variable with a no-point estimate within the zero interval is considered statistical of the sample. The bootstrapping method does not require distributional assumptions and, for this reason, has been used in ordinary least squares regression analysis to overcome non-normal distribution. The bootstrapping method involves repeatedly randomly sampling observations with replacement from the origin y significant. The indirect effects were calculated with 95% bias-corrected bootstrap confidence interval on 10.000 bootstrap samples (Model 6). Furthermore, specific indirect effects were compared through a contrast test, and the more powerful one was selected. The multiple serial mediational analysis was performed with gender as the independent variable, psychological distress as the dependent, and hours per day caring and perceived burden as serial mediators. The statistical software SPSS version 28 was used to perform the analyses, and the mediation analysis was performed with the PROCESS macro for SPSS [51].

## 3. Results

### 3.1. Gender Differences in Sociodemographic Variables, Hours per Day Caring, Perceived Burden, and Psychological Distress 

The sample included 196 (78.4%) women and 54 (21.6%) men. The caregivers’ ages ranged from 29 to 65 years (M = 42.59; SD = 6.75), and the care recipients’ ages ranged from 1 to 31 years (M = 8.46; SD = 5.28). Concerning the first analysis regarding the gender differences of caregivers, it was collected the following categorical variables of caregivers such as educational level (primary, secondary, and university studies), marital status (single/separated/widow and married/with a partner), hours per day caring (<5 h, 5–10 h, 11–15 h, and >15 h), relation with the care recipient (parents and other), and care recipient’s age (<3 years, 3–12 years, 13–17 years, and 18–31 years). As can be seen in Table 1, there were no significant differences between men and women in the sociodemographic variables. However, caregivers’ objective burden measured as hours per day caring for their relative presented significant gender differences (*χ*^2^ (3, N = 250) = 26.66; *p* =< 0.001), with half of the men caring between 5 and 10 h per day whereas half of the women were caring for more than 15 h per day. Men and women caregivers in the sample were mostly married or living with a stable partner, their education corresponded to university level, they were parents of the care recipient, and most of the care recipients were in the age interval of 3–12 years old.

For the same purpose, we added a *t*-test analysis only for the age of caregivers, which is a continuous variable. The variable caregivers’ ages were significantly different between men and women. Men (M = 46.02; SD = 7.50) were older than women (M = 41.65; SD = 4.93) (*t* (250) = 3.93; *p* < 0.001; *d* = 0.63).

Gender differences in subjective burden and psychological distress, as continuous variables, are presented in Table 2. Perceived burden and psychological distress showed significant differences, with the higher perceived burden (*t* (250) = −2.40; *p* = 0.017; *d* = 0.35) and psychological distress (*t* (250) = −2.70; *p* = 0.004; *d* = 0.40) in women than in men.

When perceived burden was analyzed as a categorical variable, the sample in this study was distributed in the four levels of perceived burden, with no caregivers in the no-burden level, 16% in the mild burden level, 50% in the moderate burden level, and 34% in the severe burden level. The percentages of women in the three levels were 10.7% in the mild level, 53.7% in the moderate level, and 35.7% in the severe level, whereas in the case of men, 35.2% were in the mild level, 37% in the moderate level and 27.8% in the severe level, showing a significant gender difference (*χ*^2^ (1, N = 250) = 18.94, *p* < 0.001), with more women in the moderate and severe levels than men. In a similar procedure that perceived burden, when psychological distress was analyzed as a categorical variable using the cut-point of ≥14 in the GHQ-12, the prevalence of psychological distress was 49.6% for the whole sample, 35.2% for men and 53.6% for women, a difference in percentages that was statistically significant *χ*^2^ (1, N = 250) = 5.73, *p* = 0.017.

### 3.2. Correlation Analysis between Caregivers’ Ages, Hours per Day Caring, Perceived Burden, and Psychological Distress for Men and Women Separately

Before performing a mediation analysis, it was important to find out the relational picture that the variables exhibit. The caregivers’ ages and the hours per day caring were added to the correlation analysis, in addition to perceived burden and psychological distress based on the gender differences shown in the previous analyses. From these four variables, only hours per day caring is a categorical one; therefore, any correlation between hours per day caring and the other variables has been performed with Spearman‘s rho. These correlations are shown in Table 3 separately for men and for women. The stronger, positive, and more significant relations are presented between perceived burden and psychological distress in both men and women. Additionally, caring for more hours per day showed a positive and significant association with perceived burden in men and women, with a higher significant level in men than in women. However, the relation between hours per day caring and psychological distress showed a different pattern for men and for women. While for men, it is positive and significant, for women, there was no relation between those variables. Finally, the caregivers ‘age showed no significant relation with the variables analyzed neither in men nor in women. 

### 3.3. Multiple Serial Mediation

**Figure 1:**
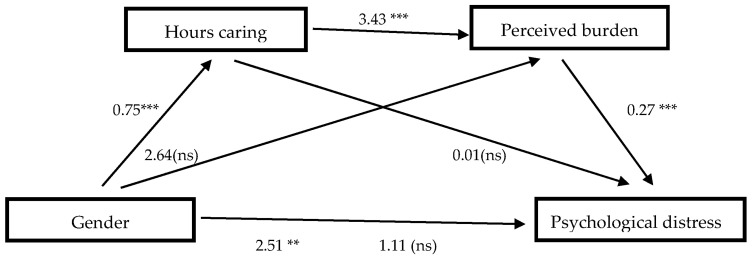
Multiple serial mediations of objective and subjective burden between gender and psychological distress. *β* values; ** = *p* < 0.01; *** = *p* < 0.001; ns = non-significant.

Figure 1 shows the mediation of burden, both objective and subjective, between gender (women/men) and psychological distress. The total effect of gender on psychological distress was statistically significant (*c* = 2.51, *SE* = 0.93, *t* = 2.70, *p* = 0.007, 95% CI [0.68, 4.35]) (Step 1). In addition, the direct effects of gender on daily hours caring was also significant (*β* = 0.75, *SE* = 0.14, *t* = 5.20, *p* ≤ 0.001, 95% CI [0.46, 1.03]), but the indirect effect of gender on perceived burden with hours per day caring as mediator was not significant (*β* = 2.64, *SE* = 2.23, *t* = 1.18, *p* = 0.237, 95% CI [−1.75, 7.03]). The direct effect of hours per day caring, as the first mediating variable, on the second mediating variable, the perceived burden, was at a significant level (*β* = 0.62, *SE* = 0.93, *t* = 3.67, *p* < 0.001, 95% CI [1.59, 5.27]) (Step 2). On the other hand, the direct effects of the mediating variables on psychological distress showed that the effects of hours a day caring were not significant (*β* = 0.01, *SE* = 0.33, *t* = 0.04, *p* = 0.972, 95% CI [−0.64, 0.67]), but the effect of perceived burden was significant (*β* = 0.27, *SE* = 0.02, *t* = 12.13, *p* ≤ 0.001, 95% CI [0.22, 0.31]) (Step 3). When gender and the mediating variables were simultaneously entered into the equation (Step 4), the relation between gender and psychological distress was not at a significant level (*c’* = 1.12, *SE* = 0.77, *t* = 1.44, *p* = 0.150, 95% CI [−0.41, 2.64]), producing a total mediation, where gender lost the relevance as psychological distress predictor, and the relevance passed to the mediating variables. The overall model was significant (*F*(3,136) = 7.29, *p* = 0.007) and explained 41% of the total variance in psychological distress.

The indirect effects and the contrasts between them are shown in Table 4. As we can see, only model three, which includes the perceived burden mediating gender on psychological distress, seems to be significant, and when the contrasts were performed, it appeared stronger. 

## 4. Discussion

The current study presents an evaluation of objective and subjective burden and psychological distress from a gender perspective in ASD caregivers. Women are overrepresented in the role of caregiver when a relative is in a dependent condition [52]. On the other hand, studies comparing men and women caregivers present results where women show higher burden and psychological distress than men [44,45,46,47,48]. 

Our results confirm the first hypothesis and clearly show that women, most of them mothers of the care recipient, dedicate more hours to caring than men, most of them fathers of the care recipient, confirming that ASD female caregivers present a more objective burden than male caregivers. This result confirms those found by Vast et al. [37] in women caring for an ASD relative who performed tasks and took responsibility inside the house, tasks that resulted more crucial and basic in the day-to-day for their care recipient relative, against the tasks performed by men which used to be outside the house, not immediate and with low involvement. The perceived burden was at a moderate level for both men and women, considering the four levels proposed by Mulud and McCarthy [20] in the Caregiver Burden Interview [19] scores. These moderate levels of burden in ASD caregivers were also found in several studies [16,17,18,21], showing that caring for an ASD relative involved an important effort and dedication in most of the caregivers. However, the perceived burden levels shown in the sample of our study are higher than those found in other studies [21,22].

Women in this work also showed a higher perceived burden than men. The higher number of hours caring for their relative could be a reason for this gender difference, but other factors could also be playing an important role, including the type of tasks performed by women, more basic and relevant to the care recipient, or the secondary stressors associated with caregiving, such as the loss of leisure time and social support, isolation, and the inability to do work outside the home, as proposed in the adaptation of Lazarus and Folkman Stress Theory [53] proposed by Pearlin et al. [54]. The higher subjective burden in women in our sample, when compared to men caring for an ASD relative, confirms similar findings in other studies of ASD caregivers [37,41].

Several studies have also shown that women caring for an ASD relative experience poorer mental health than men performing a similar task [37,40,41,42,43]. Our results confirm these findings, with women in our sample showing higher psychological distress than men. Women are at higher risk of mental problems as a consequence of the number of hours they dedicate to caring, being exposed to most of the factors associated with a worsening in their mental health, including behavior problems and co-occurring conditions in the care recipient [27,28], lack of a job outside home that can result in a lower family income [27,34,35], stress and hassle in the daily activities [25,29], and higher levels of burden [18,22,23,24,30]. The use of the GHQ-12 [49], a short screening instrument designed to detect current non-specific mental disturbance in primary care settings and in the general population, showed that half of the women caring for an ASD relative exhibited symptoms of a non-specific mental problem. The strong relationship between burden and psychological distress found in this work and the last cited studies [18,21,22,23,24] led us to the second hypothesis.

This second hypothesis is focused on the mediating role of burden between gender and psychological distress. Our results confirm the important mediating role of burden between gender and psychological distress. Gender presents a significant predictive role of psychological distress, but when burdens, both objective and subjective, are introduced into the equation, the relevance of gender falls to a non-significant level, producing a full mediation. This information shows us that the higher number of hours caring for an ASD relative and the higher perceived burden have a negative impact on mental health. There are two elements to be considered. The first one is the low relationship between hours per day caring and psychological distress in women, which suggests that the causes of the poorer mental health in women are more complex than just the high number of hours dedicated to caring. Women showed more perceived burden than men; therefore, it is necessary to find out which other factors contribute to the perceived burden that is affecting their mental health. A second result to be highlighted is the fact that the third regression model—with gender as an independent variable, perceived burden as the mediator, and psychological distress as the dependent variable—is the strongest when the three regression models are compared. This model gives perceived burden a key role in the explanation of mental health problems over the mediating role of hours per day caring (objective burden). Other conditions that can affect perceived burden and, consequently, psychological distress in women can include lower family income [27,34,35], usually when only one member of the couple is working; hassle in the daily caring tasks [25,29], more often in women for the longer time they dedicate to caring; and the behavioral problems and co-occurring circumstances in the care recipient [27,28], again with higher probability that it is the mother who copes with them due to the longer hours dedicated to caring. All these circumstances may play a role in explaining the worse mental health shown by women caring for an ASD relative in comparison with men.

The results of the present study represent a valid contribution to the knowledge of the mental health of family caregivers of ASD people, highlighting the importance of burden as one of the variables more relevant for its role as a mediator between the time dedicated to caring and psychological distress. ASD caregivers face challenges related to their care recipients, such as health care and education, besides those affecting their own lives as a consequence of their dedication to caring, including social life, financial issues, and emotional problems [12,13,14]. All these aspects could have a role in the caregiver’s perceived burden [15]. Future research should focus on the factors that affect subjective burdens, specifically in women, beyond the number of hours dedicated to caring. This would include any co-occurring circumstances that might affect ASD caregivers’ burden, such as also caring for an elderly or disabled family member, other underage children, or the parents being divorced.

The present work has limitations. The generalization of results from this study must consider the sample characteristics, being composed mostly of mothers who are married or living with a stable partner and have undertaken university studies. Also, the information has been obtained from self-reports that could be affected by bias such as social desirability. Finally, the cross-sectional design used does not allow causal relationships, and the use of a convenience sample, even having participants from most of the Spanish provinces, could not be representative enough.

## 5. Conclusions

Our results represent an advance in the study of factors that affect the mental health of caregivers of an ASD relative and highlight gender and burden as relevant variables. Women present a higher burden than men in its two dimensions: objective, caring for more hours, and subjective, with a higher perception of burden. This study shows the relevance of burden in the levels of psychological distress in ASD caregivers, pointing it out as an obvious variable to be targeted in intervention programs for these caregivers. Time dedicated to the care of a child with ASD should be balanced between the parents, avoiding the current situation in which women, and specifically mothers, bear the brunt of the effort. Women should also receive support and psychological interventions to control the perceived burden and indirectly improve their mental health. 

## Figures and Tables

**Table 1:** Sociodemographic variables and objective burden in men and women.

	Men		Women		*χ*^2^ Value
Variables	N	%	N	%	(*p*)
Education level					0.25 (0.882)
Primary	8	14.8	25	12.7	
Secondary	20	37.1	70	35.7	
University	26	48.1	16	51.6	
Marital status					0.11 (0.746)
Single/separated/widow	6	11.1	25	12.8	
Married/with partner	48	85.9	171	87.2	
Hours/day caring					26.66 (<0.001)
<5 h	6	11.1	10	5.1	
5–10 h	28	51.9	43	21.9	
11–15 h	11	20.4	46	23.5	
>15 h	9	16.7	97	49.5	
Relation with the ASD relative					2.21 (0.531)
Parents	53	98.1	187	95.4	
Other	1	1.9	9	4.5	
Care recipient’s age					2.25 (0.522)
<3 years	0	0.0	4	2.0	
3–12 years	42	77.8	157	80.1	
13–17 years	6	11.1	22	11.2	
18–31 years	6	11.1	13	6.6	

**Table 2:** Gender differences in subjective burden and psychological distress.

		N	M	SD	*t*	*d*
Perceived Burden	Men	54	50.48	15.72		
	Women	196	55.68	13.64	−2.40 *	−0.35
Psychological Distress	Men	54	12.37	6.40		
	Women	196	14.88	5.95	−2.70 **	−0.40

* = *p* ≤ 0.05; ** = *p* ≤ 0.01.

**Table 3:** Relationships between the variables for men (left lower part) and women (right upper part, in bold).

	1	2	3	4
1. Caregivers’ ages	---	**−0.09**	**0.05**	**0.02**
2. Hours/day caring +	0.02	---	0.15 *	**0.03**
3. Perceived burden	0.15	0.42 **	---	0.59 ***
4. Psychological distress	0.16	0.51 ***	0.71 ***	---

+ = Spearman’s rho; * = *p* ≤ 0.05; ** = *p* ≤ 0.01; *** = *p* ≤ 0.001.

**Table 4:** Indirect effects and comparison between them.

			Bootstrapping 95% Confidence Interval	
Effects	Point Estimate	*SE*	Lower	Upper
1. Gender–hours/day caring–psychological distress	0.01	0.26	−0.52	0.49
2. Gender–hours/day caring–perceived burden–psychological distress	0.70	0.61	−0.45	1.94
3. Gender–perceived burden–psychological distress	0.68	0.20	0.31	1.10
**Contrasts**				
Model 1 versus Model 2	−0.70	0.72	−2.17	0.67
Model 1 versus Model 3	−0.68	0.32	−1.35	−0.11
Model 2 versus Model 3	0.02	0.66	−1.22	1.35

N = 250; k = 10,000; *SE* = Standard Error. Model 1 = gender–hours/day caring–psychological distress; Model 2 = gender–hours/day caring–perceived burden–psychological distress; Model 3 = gender–perceived burden–psychological distress.

## Data Availability

The datasets generated and analyzed during the current study are available from the corresponding author upon reasonable request.
